# Effectiveness and cost-effectiveness of embedded simulation in occupational therapy clinical practice education: study protocol for a randomised controlled trial

**DOI:** 10.1186/s13063-017-2087-0

**Published:** 2017-07-21

**Authors:** Christine Imms, Eli Mang Yee Chu, Stephen Guinea, Loretta Sheppard, Elspeth Froude, Rob Carter, Susan Darzins, Samantha Ashby, Susan Gilbert-Hunt, Nigel Gribble, Kelli Nicola-Richmond, Merrolee Penman, Elena Gospodarevskaya, Erin Mathieu, Mark Symmons

**Affiliations:** 1 Australian Catholic University, 17-29 Young Street, Fitzroy, 3065 Australia; 2 Australian Catholic University, 33 Berry Street, North Sydney, 2060 Australia; 3 Deakin University, 221 Burwood Highway, Burwood, 3125 Australia; 4 University of Newcastle, University Drive, Callaghan, 2308 Australia; 5 University of South Australia, Adelaide, 5001 Australia; 6 Curtin University, Perth, 6845 Australia; 7 Deakin University, Geelong, 3220 Australia; 8 University of Sydney, 75 East Street, Lidcombe, 2140 Australia; 9 University of Sydney, Edward Ford Building, Camperdown, 2006 Australia

**Keywords:** Simulation, Occupational therapy, Clinical placement, Efficiency, Cost, Clinical reasoning, Trial, Evaluation, Education, Simulated clinical placement

## Abstract

**Background:**

Clinical placements are a critical component of the training for health professionals such as occupational therapists. However, with growing student enrolments in professional education courses and workload pressures on practitioners, it is increasingly difficult to find sufficient, suitable placements that satisfy program accreditation requirements. The professional accrediting body for occupational therapy in Australia allows up to 200 of the mandatory 1000 clinical placement hours to be completed via simulation activities, but evidence of effectiveness and efficiency for student learning outcomes is lacking. Increasingly placement providers charge a fee to host students, leading educators to consider whether providing an internal program might be a feasible alternative for a portion of placement hours. Economic analysis of the incremental costs and benefits of providing a traditional versus simulated placement is required to inform decision-making.

**Methods/design:**

This study is a pragmatic, non-inferiority, single-blind, multicentre, two-group randomised controlled trial (RCT) with an embedded economic analysis. The RCT will compare a block of 40 hours of simulated placement (intervention) with a 40-hour block of traditional placement (comparator), with a focus on student learning outcomes and delivery costs. Six universities will instigate the educational intervention within their respective occupational therapy courses, randomly assigning their cohort of students (1:1 allocation) to the simulated or traditional clinical placements. The primary outcome is achievement of professional behaviours (e.g. communication, clinical reasoning) as assessed by a post-placement written examination. Secondary outcomes include proportions passing the placement assessed using the Student Practice Evaluation Form-Revised, changes in student confidence pre-/post-placement, student and educator evaluation of the placement experience and cost-effectiveness of simulated versus traditional clinical placements. Comprehensive cost data will be collected for both the simulated and traditional placement programs at each site for economic evaluation.

**Discussion:**

Use of simulation in health-related fields like occupational therapy is common, but these activities usually relate to brief opportunities for isolated skill development. The simulated clinical placement evaluated in this trial is less common because it encapsulates a 5-day block of integrated activities, designed and delivered in a manner intended to emulate best-practice placement experiences. The planned study is rare due to inclusion of an economic analysis that aims to provide valuable information about the relationship between costs and outcomes across participating sites.

**Trial registration:**

Australian New Zealand Clinical Trials Registry, ACTRN12616001339448. Registered 26 September 2016.

**Electronic supplementary material:**

The online version of this article (doi:10.1186/s13063-017-2087-0) contains supplementary material, which is available to authorized users.

## Background

Clinical placements are integral to occupational therapy professional education. Theoretical knowledge derived from formal education needs to be supported by exposure to the practice environment for learning to be maximised [1–3]. Through immersion in workplace learning, clinical placements provide students with opportunities to synthesise theoretical and tacit knowledge to develop professional competencies [4, 5]. Fundamental elements of placements for any professional academic program provide students with opportunities to apply theoretical knowledge and practical skills developed during their course as well as engage in real-world practice and decision-making and in the role they are learning to become [6–9].

Varied terms have been used to describe clinical placements in the literature. For example, the World Federation of Occupational Therapists [10] uses the terms *fieldwork education* or *practice education* to refer to the professional practice placement component of educating student occupational therapists. Other terms include *clinical education* and *preceptorship*. In this paper the term *clinical placement* will be used in two ways as follows:

- *Traditional clinical placement (TCP)* refers to the clinical placement experience that occupational therapy students undertake for their clinical education within a professional setting that provides services to the community. Such settings include hospitals, schools, private practices and various other for-profit and not-for-profit organisations.
- *Simulated clinical placement (SCP)* refers to a collection of planned and integrated activities that aim to emulate a traditional clinical placement, in which the environment, people, materials, activities and processes of work are simulated to create a facsimile of occupational therapy practice.

Occupational therapy programs must adhere to the minimum standards for clinical placements specified by the World Federation of Occupational Therapists [10] and the Occupational Therapy Council (Australia & New Zealand) Ltd (OTC) for accreditation. Students must complete a minimum of 1000 hours of clinical placement during their degree [11]. However, universities find it increasingly difficult to source a sufficient number of quality clinical placements to provide all students with the opportunity to practise and demonstrate key clinical skills and professional competencies across a range of practice areas [12, 13]. This situation arises from a number of pressures associated with changes in work practices and organisational structures in healthcare, as well as programs on offer and the students who enrol in them. Specific examples of these pressures include the following: a shift of healthcare services from institutions to community settings; increased part-time work for supervisors, allowing less time for student supervision; more emphasis on risk management in patient care; and growing numbers of university occupational therapy training programs and more students enrolling in them [13–15].

Innovative teaching and learning strategies are required to ensure students are provided with quality clinical placements throughout their professional education. To meet these learning needs, simulation-based learning has been utilised as an educational strategy in medicine [16], nursing [17–19] and allied health [20]. A project conducted in 2010 by a team from the University of Queensland (UQ) on behalf of Health Workforce Australia examined the use of simulation in occupational therapy curricula [21]. The project reviewed all Australian occupational therapy programs and identified only three that utilised simulation-based learning to replace any part of a clinical placement. These three programs counted between 5 and 15 hours of simulation-based learning towards the 1000 hours of required placement. Rodger et al. [9] identified several main barriers to implementing a sustainable simulation-based learning program. These barriers included a lack of resources such as simulated environments, limited access to standardised (simulated) patients and issues of sustainability related to recurrent funding and training needs.

The potential for simulation-based learning activities to replace some clinical placement hours to increase placement capacity and improve readiness of students to commence clinical placements was identified [21]. A key outcome of the project was the development of a consensus statement regarding the extent and quality criteria for simulation-based learning. This consensus stated that up to 20% of the required 1000 hours of clinical placement could be delivered via simulation activities, provided the following design and delivery conditions were met:

- High level of authenticity for occupational therapy practice
- High level of complexity requiring student engagement and interaction
- Immediacy to interaction with a real client and to occupational therapy clinical placements
- Assessed with respect to meeting occupational therapy clinical placement objectives
- No one simulation modality to be used as a ‘stand-alone’ alternative to clinical training time ([21], p. 6)

Following public consultation, the OTC [11] finalised new accreditation standards for Australian occupational therapy programs of study, and these were approved by the Occupational Therapy Board of Australia in 2013. The approved standards stipulated that up to 20% of a minimum of 1000 hours of clinical placement (200 hours) may include well-designed simulation-based learning activities. Published explanatory guidelines elaborated the standard requirements for simulation when used for clinical placement. The guidelines identified the potential for simulation activities to be used to ensure students are adequately prepared to commence clinical placement, to ensure students achieve threshold competencies and safe practice and to enhance learning and reasoning in follow-up to clinical placement. Additionally, the guidelines stated that simulation-based learning activities that contribute to clinical placement hours must meet the five criteria identified previously [21]. Importantly, the OTC [11] guidelines stipulated that student interaction with a real client may be with a standardised (simulated) patient. This provides the opportunity for simulation to be used as a full substitute for up to 200 of the required 1000 clinical placement hours, rather than only to augment clinical placement experiences.

Few studies have evaluated the effectiveness of using simulation to substitute TCPs in allied health. In one study [22], randomised controlled trials (RCTs) were used to investigate whether simulated learning environments (SLEs) for physiotherapy can, in part, substitute for a TCP experience. A simulation program was developed to replicate clinical placement in musculoskeletal practice in an ambulatory care setting to replace 1 week of a 4-week clinical placement. Three hundred and seventy physiotherapy students from six universities in Australia participated in an RCT. It was concluded that students’ achievement of clinical competencies was equivalent between the SLE and TCP groups.

Two independent, parallel, single-blinded, multicentre RCTs were conducted [23] to compare physiotherapy students’ achievement of clinical competencies in SLEs against traditional cardiorespiratory clinical placement. Students participated in practice learning in a traditional ward setting or a simulated ward environment. Either a simulated patient or a high fidelity simulator was used in the simulated ward. A total of 349 physiotherapy students from seven universities participated in these trials. Student performance was comparable between the SLE groups and the TCP groups. Students were also satisfied with their simulation learning experience. Evidence from this study supported the notion that simulated clinical experiences can replace part of a clinical placement and achieve no worse outcomes. The work of Watson et al. [22], Blackstock et al. [23] and an unpublished simulation pilot program at Australian Catholic University in occupational therapy suggest that simulation-based learning can be an effective alternative to TCP in allied health professional education. However, further empirical evaluation is needed.

There is a body of literature describing the benefits of simulation for training and education, though rigorous evaluations are more sparse [24]. There is evidence to indicate benefit in using simulated clients/patients for fostering patient-centred skills for students in nursing [25] and psychiatry [26]; that simulation use can improve knowledge, skills and attitudes as part of a larger program for physical therapy students [24]; and that it is beneficial for specific medical interventions such as resuscitation [27]. A comprehensive systematic review and meta-analysis [28] found small but positive overall benefits for simulation for students in medicine, nursing, dentistry, emergency medicine and other medical fields, but those benefits were associated with a higher cost. However, a longitudinal study [29] found no skill-based benefit amongst nursing students who had undertaken the simulation curriculum, and a large longitudinal study involving nursing students [30] found no difference at the time of final assessment after replacing either 25% or 50% of traditional clinical hours with simulation versus a control group.

Applying the knowledge gained from prior research to occupational therapy programs needs to be considered in light of the technologies and techniques used in the healthcare simulation field, which are often specific to discipline practices and constantly evolving. Thus published research can date quickly or be difficult to generalise. There is also significant variation in what is meant by the term *simulation*. Learning activities can be defined as simulation when students watch video footage of procedures being demonstrated, or actors are hired to play the role of patient/client or when students engage with an interactive computer-based activity or medical device. Simulation is also the term used when a fully immersive virtual reality experience is provided. What outcome is studied in research also varies, increasing the complexity of interpreting and using the findings. Thus carefully conducted empirical studies, with clearly defined procedures, and systematic reviews of research are of the greatest value for informing educational practices. In addition, education providers need to make decisions about resources that are fiscally responsible. It is possible that simulation may be able to provide an effective learning environment for some student outcomes, but the cost of achieving this is not known.

The purpose of the current project is to compare the learning outcomes between those students who undertake an SCP and those students who undertake a TCP, and to assess the costs of developing and maintaining the SCP compared to sourcing and implementing TCPs. The study will focus on students in the earlier years of their occupational therapy degree programs and on determining whether students who experience an SCP achieve non-inferior outcomes compared to those who experience a TCP. Efficacy, cost-effectiveness and affordability results will inform universities considering these placement activities.

## Methods

### Design

**Fig. 1:**
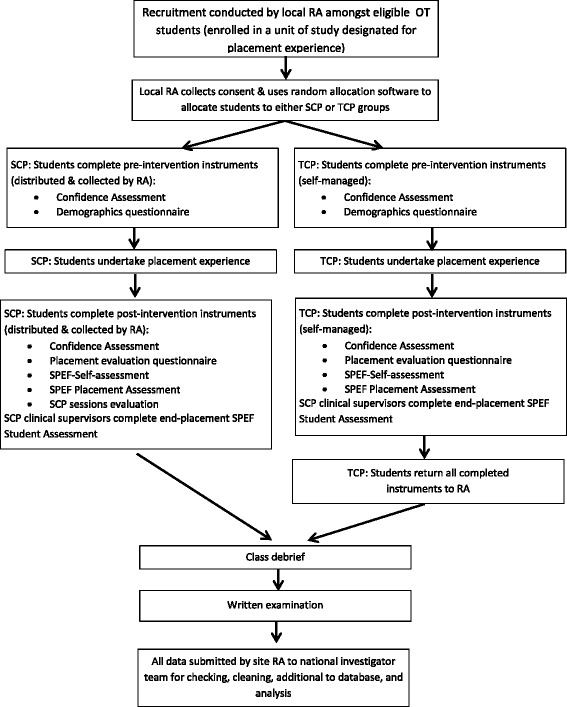
Flow diagram of the RCT of simulated clinical placement (*SCP*) versus traditional clinical placement (*TCP*) programs

**Fig. 2:**
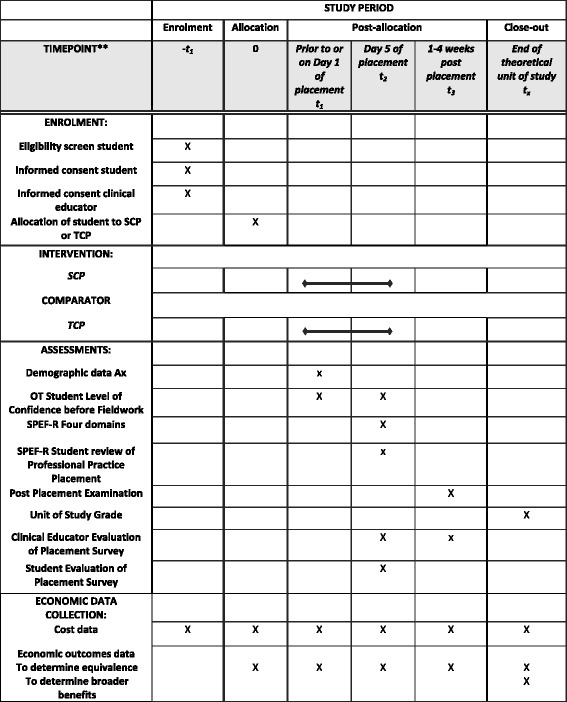
SPIRIT chart for stages of assessment in this RCT. A populated SPIRIT checklist is available in Additional file 1

The study is a pragmatic, non-inferiority, single-blind, multicentre, two-group RCT with an embedded economic analysis. Figures 1 and 2, respectively, display a flowchart and a Standard Protocol Items: Recommendations for Interventional Trials (SPIRIT) chart of assessment data for the study. A populated SPIRIT checklist is provided in Additional file 1.

### Aims and research questions

The study aims to explore the differences between a 40-hour block SCP experience and a 40-hour block TCP experience in terms of development of professional competencies and fieldwork confidence, and to determine the comparative delivery costs for the SCP and TCP programs as borne by the university.

The research questions are:

1. Do students who undertake an SCP in the early years of an entry-level occupational therapy program achieve *non-inferior outcomes* in their development of professional practice skills compared with those who attend a TCP, as assessed by a post-placement written examination (primary outcome), Student Practice Evaluation Form-Revised (SPEF-R) evaluation [31, 32] and end-of-semester study unit score?
2. Do students and supervisors report similar professional practice learning opportunities when students complete an SCP compared to those students who complete a TCP, assessed using a self-report survey, and similar frequencies of *not applicable* and *insufficient observation* items in the SPEF-R?
3. Economic research questions:
  If non-inferiority is established, does SCP cost less than TCP from the perspective of (1) the health sector and (2) the universities as providers?
  If costs of SCP are not less than those of TCP, are there offsetting benefits which still make the SCP value for money?
4. Do students who experience an SCP in the early years of an entry-level occupational therapy program report self-confidence outcomes that are non-inferior to those who attend a TCP, as measured by the Student Confidence Questionnaire [33]?
5. What are the perceptions of quality and adequacy of the TCP versus SCP experience of students and clinical educators?

### Location and timing

The multicentre trial will be coordinated from one university by a national research team. It will be delivered, and separately managed, at six universities by local implementation teams. One of those universities will implement the trial at three campuses; thus the trial will be implemented at eight different sites. The clinical placement experience will be integrated into a unit of study within occupational therapy courses at each university at a time during 2016 deemed most appropriate by each course convenor. Thus, while the placement block (SCP and TCP) occurs across a consistent and intensive 5-day period, the timing of delivery of that block is at the discretion of each course convenor to ensure best fit with each university’s individual curriculum and any other local considerations.

In some cases the trial placement program replaces existing placement hours within the course, and in others the course convenor may elect to add the intensive week to existing placement arrangements. Any modifications to curricula that are required to embed the placement experience into the units of study will be made by course convenors and subject to appropriate university approvals obtained prior to implementation. Due to the geographical distribution of sites across Australia and the different systems employed at each university, each site will have its own local implementation team that includes a site coordinator, various academic and clinical staff and a local research assistant. The economic appraisal is coordinated and conducted across all sites by one economic team.

### Participants

All students enrolled in the chosen units at each participating university are required to complete the placement program (TCP or SCP) as part of their studies and are eligible to participate. All of these students are also required to complete the various instruments used to collect data for the study, some of which form part of the assessment for their studies. Participation in the research (as opposed to participation in the placement program) involves students consenting for their data to be included in the trial. Data from students who do not consent to participate will be used for local assessment purposes where relevant, and destroyed where not relevant. Only data from consenting students will be submitted to the investigator team. Participation in the RCT study is voluntary. Students who are repeating the unit of study are ineligible to participate in the trial (but may still complete the unit of study and the placement program). There are no other exclusion criteria. Along with student participants, clinical supervisors and educators involved in the delivery of the placement program (TCP and SCP) are eligible to participate. Their participation is voluntary, and informed consent will be obtained.

#### Sample size

The SCP has been trialled at the managing university within a second-year level unit of study in an undergraduate occupational therapy bachelor degree. This provided a set of indicative student outcomes allowing for calculation of a sample size estimate. The primary outcome used for this calculation was the post-placement examination score (range 0–100%). Non-inferiority was defined as a mean score difference in exam scores between SCP and TCP groups of ≤7%. This value was based on the standard deviation of student examination scores obtained within the unit of study where the SCP was piloted in 2013.

Using standard power estimate criteria (to achieve a power of at least 0.8, alpha level 0.05), 120 students per group are required (total sample = 240). To account for clustering according to university site, where an average cluster size of 50–60 students is anticipated, and an estimated intraclass correlation coefficient (ICC) of 0.007, an additional 100 students will be required (*n* = 340). Finally, to allow for a 20% attrition rate (340/0.8), a total sample of 425 participants is required.

#### Recruitment

Eligible occupational therapy students will be provided with information about the study and invited to participate at the beginning of, or just prior to, the start of the semester in which the trial is to be implemented. All students will be informed that (1) if enrolled in the unit they will undertake either the SCP or TCP as determined by random selection; (2) all students will complete the same instruments towards their assessment and provide feedback on their placement experience; and (3) only data from students who consent to participate will be included in the trial. Students will be told that taking part in the trial and allowing their data to be used is voluntary and that their experience and grades in the unit of study will not be affected should they choose not to participate. Prior experience suggests the recruitment rate of students (with efficient and focussed strategies) will achieve 85–90% of the student population. With multicentre trials, the rate is often less, perhaps 70%, although Watson et al. [22] found that, of 1200 eligible students for a study using simulation to replace clinical time, only 410 students volunteered to participate (34%). Therefore we estimate that a recruitment rate of 60% of eligible students will be achievable. In 2015, the participating universities estimated that 774 students would be eligible for inclusion in the trial in 2016, thus a sample of 425 (55% of the expected eligible sample) appears feasible.

### Randomisation and allocation

Using a procedure specified by the national research team, the local research assistant at each site will recruit students to the trial, collect consent forms and allocate students to either the SCP or TCP group in a 1:1 ratio. A software package will be used to generate the random allocation sequence of students who have consented to submit their data for the trial. This software is freely available from the Internet (http://random-allocation-software.software.informer.com/2.0/) and is described in the literature [34]. The allocation is performed at each site by the local research assistant utilising block randomisation once student enrolment in the unit of study is determined.

### The SCP intervention

The SCP, called *OTSimPrac*, aims to fully substitute 40 hours (a 5-day working week) of TCP. It is designed to be situated in the foundation years of an occupational therapy program where the focus of learning is on development of professional behaviours, self-management, communication and information-gathering skills that are applicable in all placement or work settings. The SCP is designed to address criteria stipulated by the Occupational Therapy Council for simulation-based placement with the aim of being eligible to contribute to the 1000 hours of clinical placement mandated within the Occupational Therapy Accreditation Standards [11]. Examples of how these five criteria are addressed are provided in Table 1.

**Table 1:** Design features of the SCP in relation to the five criteria set by Rodger et al. (2010) [21]

Design criteria	Operationalised in the SCP by:
1. High level of authenticity for occupational therapy (OT) practice	• Development of authentic case studies and validation of case studies by external healthcare professionals • Setting the pace and duration of the SCP to reflect a realistic timeframe for the actual clinical processes • Conducting simulated clinical activities in authentic environments (simulated or actual practice environments) • Providing opportunity for the students to learn through observation/role modelling and by working alongside each other • Including and assessing a breadth of skills, knowledge and attributes rather than isolated or de-contextualised components • Requiring individual- and group-based activity engagement by each student • Requiring students to interview and communicate with clients, healthcare professionals and other stakeholders (e.g. employers of clients in a vocational rehabilitation setting) within the client intervention process • Using authentic clinical/professional tools and processes in developing rehabilitation/intervention plans • Requiring students to communicate assessment results and intervention recommendations with clients and key stakeholders via clinical conferences, presentations and written reports and/or other professional documentation • Providing students with feedback from clinical supervisors and simulated clients and other stakeholders (e.g. employers) about the quality of the rehabilitation or intervention plan, professional behaviours, communication and self-management
2. High level of complexity requiring student engagement and interaction	
3. Delivered with immediacy to interaction with a real client (may be portrayed by a standardised patient) and to OT clinical placements	• Use of standardised patients/actors and practicing health professionals to enable students to interact with ‘real’ clients and professionals in the simulated practice setting • Supervision of students by practising occupational therapists in the field and tutors with professional practice skills relevant to the SCP
4. Designed and assessed to meet OT clinical placement objectives	• Specific placement objectives and learning outcomes are pertinent to the achievement of foundational clinical practice skills • The learning focus of this SCP is for students to develop core professional practice behaviours and skills. These core professional behaviours and skills are pertinent to all clinical practice areas and include *(1) professional behaviour, (2) self-management skills, (3) co-worker communication and (4) communication skills*
5. No one simulation modality can be used as a ‘stand-alone’ alternative to clinical training time	• Opportunity for students to learn through observation, role modelling and by working alongside each other whilst engaging with a variety of simulation modalities. Modalities will include use of written and video case material, standardised clients, actors and mock (role play) clinical case conferences

The structure and activities of the SCP are based on the Canadian Model of Occupational Performance and Engagement and the Canadian Practice Process Framework [35] to enhance applicability and transferability to various occupational therapy programs. The model and framework are used both nationally and internationally by occupational therapy degree programs to guide occupational therapy students’ clinical reasoning and to enable them to focus on person-centred, occupationally based professional practice. For this trial, materials have been developed to support the SCP, targeting three types of units of study within occupational therapy degree programs: vocational rehabilitation, adult mental health and adult physical rehabilitation. Each of these three *streams* of occupational therapy practice will be part of the trial, with each site able to choose the *stream* that best suits their occupational therapy program. This feature will serve to increase generalisability of the results.

A range of detailed case scenarios have been developed for the three settings, including realistic and comprehensive case file notes and briefing documents that describe clients who require occupational therapy services within one of the three targeted settings. Steps were taken to optimise immersion in the SCP as part of the design process. For instance, university rooms and other spaces used for SCP will be branded with a fictitious placement provider name. A mock corporate placement intranet website with no university branding has been established to host resources and information for students and the staff involved. Client-related forms and referral documents were designed to emulate what might be used by occupational therapy providers and were branded with a logo. To add to the authenticity of the SCP placement, external occupational therapy professionals practising in the relevant field will be employed to provide supervision of the SCP students and to assess students’ learning outcomes.

Implementation of the 40-hour SCP intervention is structured as follows:

1. Students will work with practising clinical supervisors and simulation facilitators in small groups with an SCP supervisor-to-student ratio of 1 to 9 or 10.
2. Tutorial rooms and small offices on the university campus, or off campus if such facilities are available, are allocated for the placement as working spaces for each of the simulated placement days.
3. An induction program is provided on the first day to introduce students to the workplace, supervisor(s), placement structure and content, work processes and expected professional behaviours.
4. Each student will work with two case scenarios in particular, one in significant depth involving a simulation patient, and they will also be exposed to a further two or more cases during the 40-hour SCP
5. Students will work in small groups (with an average of three students per group, ranging from two to four students) to complete a series of simulation activities pertaining to their primary case scenario involving:
  - Interactions with a standardised client (simulated patient/actor) to conduct an interview and conduct an activity analysis based on observation of relevant task performance (e.g. preparing lunch or engaging in a workplace task)
  - Engagement with a health professional (e.g. a general practitioner or a social worker) and other relevant stakeholders (e.g. a family member) in the client’s life by telephone
  - An assessment of an appropriate external environment such as a workplace, shopping centre or home.
  The processes of clinical practice will include initial assessment, interviewing relevant stakeholders, intervention planning, case conferences and documentation. The simulation is designed to replicate real-time, complex and sequential activities as they occur in actual professional practice. Students will work in larger groups for some learning activities.
6. Students will be provided with written documentation such as a client referral, shown a video of a client interview conducted by a professional occupational therapist and provided with a realistic case file document for a simulated client on which to base their own documentation. Students gather clinical information related to a primary client (the simulation patient/client they interview and observe), write up assessment findings, keep daily progress notes and develop an intervention plan for the client.
7. In addition to their active involvement with a simulation client, students will be exposed to further (secondary) cases by responding to a referral of a second client and attending simulated case conferences and case presentations led by other students.
8. Students receive feedback from simulated clients, health professionals, clinical supervisors and teaching staff as the SCP progresses.
9. SCP students incorporate all of the information gathered through the week to prepare a comprehensive file on their client and present intervention recommendations at a case conference held on the final day.

Standardisation of the intervention is supported by a set of resources, explanatory notes, activity guides and other materials, which are incorporated into a set of delivery manuals distributed to each of the eight participating sites. During the development phase, occupational therapy staff members from each partner university, and representing each of the three clinical practice areas (vocational, mental health, physical rehabilitation), contributed to the curriculum through a committee structure to ensure relevance, applicability and authenticity of the materials developed. The manuals are to be adhered to at each site to maximise consistency of SCP delivery. A training officer will provide comprehensive briefings to academics and supervisors at each trial site prior to implementation. This information will also be used to brief professional actors to take on the roles of standardised/simulated clients.

### The TCP comparison group

The TCP will match the SCP in terms of duration (40 hours over 5 days), timing (implemented at the same time or within 1 week of each other), evaluation and setting (vocational rehabilitation, mental health or community-based physical rehabilitation). Students will be placed as individuals or in pairs, according to the needs and limitations of the particular external placement provider.

The learning outcomes for the clinical placement program are the same for the SCP and TCP students and will be communicated to them and the clinical educators prior to placement. The overarching learning outcomes are that students will develop and demonstrate behaviours appropriate to the occupational therapy profession, including:

1. Professional self-conduct
2. Self-management skills (i.e. effective time management, assuming responsibility for own learning, demonstrating initiative and taking responsibility for own actions and responses to supervision and constructive feedback)
3. Effective communication with co-workers and service users and ability to work as part of a team
4. Skills in effective client information gathering from a range of sources
5. Ability to identify a client’s occupational performance issues and assess the impact of environmental and social factors on the client’s ongoing participation and engagement

**Fig. 3:**
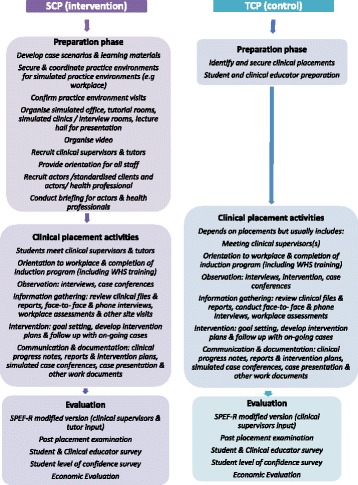
Comparison between the SCP and TCP intervention pathways

A comparison of the SCP and TCP pathways is presented in Fig. 3.

### Instruments and measures

Outcomes will be assessed using a variety of tools to gather both qualitative and quantitative data to address the research questions. Data will be collected immediately prior to or at the beginning of placement, on completion of placement and at the end of the unit of study (see Table 2). Demographic data including student age, gender, university, prior qualifications and prior placement experience will also be collected.

**Table 2:** Description of the instruments used to collect data across the trial

Instrument	Type	Timing	Respondent
SPEF-R	Paper-based	Complete across placement week, finalising during morning of 5^th^ day of placement	Clinical supervisors
SPEF-R, self assessment	Paper-based	Complete across placement week, finalising during the 5^th^ day of placement	Students (self-report)
Pre-placement questionnaire	Paper-based	Pre-placement briefing 1 week prior to placement *Or* morning of 1^st^ day of placement	Students (self-report)
Post-placement questionnaire	Paper-based	Afternoon of 5^th^ day of placement	Students (self-report)
SPEF-R, student review	Paper-based	Complete across placement week, finalising during the afternoon of 5^th^ day of placement	Students
Examination	Paper-based	Within 4 weeks of placement conclusion	Students
Sessions evaluation	Online	Within 2 weeks of placement conclusion	SCP students only
Focus group feedback	Transcribed audio	Within 4 weeks of placement conclusion	Students Clinical educators
Clinical educator survey	Online	Within 2 weeks of placement conclusion	Clinical educators (clinical supervisors and simulation facilitators)
Cost data collection	Digital	Across the full extent of the trial	Partner university site coordinators, investigators, placement support staff and project support staff

*SPEF-R* Student Practice Evaluation Form-Revised

#### Written examination (primary outcome)

Whether students who attend the SCP achieve non-inferior outcomes to those attending a TCP in development of professional skills will be determined according to the results of an end-of-placement examination. The written examination will be implemented 1–4 weeks after placement is completed. It will require students to use (1) reasoning skills to integrate knowledge and professional skills relevant to the broad context of the theoretical unit and placement experience (i.e. vocational rehabilitation, mental health or community rehabilitation) and (2) expected professional competencies commensurate to the students’ stage of professional development. The examination will constitute a series of questions based on a scenario-based clinical report provided at the beginning of test. The aim is to test students’ skills in gathering and integrating pertinent information about the client; their capacity to identify relevant occupational performance issues and capacities and environmental barriers to performance; and their skills in identifying further information, sources and methods that could be used to gather information relevant to the client in the scenario.

The examination will be scored using a continuous scale, ranging from 0 to 100%. The examinations will be graded by a panel of clinical educators who will be blinded to the placement type undertaken by each student. They cannot be reliably blinded to university of origin because of the distributed timing of implementation. The panel will use an examination-specific marking rubric [36] and be trained through a series of workshop sessions involving grading a number of examinations and comparing and discussing the relative results. An inter-rater reliability assessment will be employed to track the readiness of each member of the panel to grade student examinations. An ICC of at least 0.7 will indicate acceptable reliability.

#### Student Practice Evaluation Form-Revised

Whether students pass their placement will be assessed using the Student Practice Evaluation Form-Revised (SPEF-R). The SPEF-R is a standardised and validated instrument used to assess professional competencies of occupational therapy students [31]. It is routinely used across Australia and is completed by a clinical supervisor to assess students’ practice competencies. The SPEF-R was first developed in 2004 and revised in 2011, and further validation research has been undertaken to establish the psychometric properties of the tool [32]. The SPEF-R is suitable for evaluating students in a wide range of placement settings, including health (acute, rehabilitation and community services including physical medicine and mental health) and education, and for placements involving paediatric or adult clients. It allows for the assessment of a broad range of skills and competencies.

Student performance is usually evaluated across eight domains in the SPEF-R; however, only the first four will be used in this study. Use of an approved modified version of the SPEF-R is common when assessing students in foundation/early years of their programs, as the placement hours are often fewer (e.g. 1 week rather than 6 weeks) and the competencies required of students more limited. The four domains of interest in this trial are:

1. Professional behaviour: *conducts self in a professional manner*, assessed using five items, of which four are core items that must be passed
2. Self-management skills: *demonstrates effective self-management skill,* assessed using five items, of which two are core and a further two are required to achieve a pass in the domain
3. Co-worker communication: *communicates effectively within the workplace,* assessed using three items, of which two are core and must be passed
4. Communication: *communicates effectively with service users and significant others,* assessed using five items of which four core items must be passed

Each SCP and TCP student will be evaluated by their clinical supervisor across the four SPEF-R domains of interest. Each student will also complete a matching SPEF-R in a reflective, self-assessment mode, before receiving final feedback from their clinical supervisor. The SPEF-R allows for the inclusion of written comments. Assessors of student performance using the SPEF-R cannot be blinded to group allocation of the student, as they must have observed the student on placement.

Individual student performance on each item is rated on a 5-point scale as follows: 1. *Performs unacceptably*; 2. *Experiencing difficulty or performs marginally*; 3. *Performs adequately*; 4. *Performs proficiently*; and 5. *Performs with distinction*. Scores of 1 or 2 indicate a failing performance. Students must pass all core units in each of the four domains of the SPEF-R to achieve an overall pass in the placement. Data will be collated to determine whether students pass overall, which domains are passed and the sum of the individual item scores in the SPEF-R. The frequencies of items identified as *not applicable* and *insufficient observation* in the SPEF-R will also be determined for each student. The student and supervisor reports of comparable learning experiences in each placement setting will be compared using data extracted from the SPEF-R and a self-report survey (research question 4).

#### Unit grade

The overall grade for the unit of study will be collected for each participating student and will be reported using a continuous scale ranging from 0 to 100%. A mean difference of ≥7 points on the subject/unit grade between groups will be considered an educationally meaningful difference. This difference was selected as meaningful because it represents ≥50% of each passing grade scale (e.g. Pass = 50–65% in some universities and 50–60% in others), and a difference of 7 points is likely to result in a shift in the allocated grade for a student.

#### Economic data collection

To assess cost-effectiveness of SCP in comparison to TCP, data on resource use will be collected. For SCP the opportunity cost of accommodation (e.g. simulated office, tutorial rooms) and other capital costs (e.g. equipment, databases developed specifically for simulation practices), repair and maintenance will be collected. Staff time will be estimated with information about activities undertaken to deliver both the SCP and TCP. Activities that are undertaken before the placement, including time used in preparation for the SCP and time used to secure a TCP, will be included. Time spent on these activities will be multiplied by the corresponding hourly rates and adjusted for overheads. The cost of developing the SCP materials and financial reimbursement to TCP providers will also be collected. Purpose-built cost templates will be used to collect these data from the appropriate sources within each university.

#### Student confidence

Student confidence will be measured prior to placement and again at the end of placement using the Student Level of Confidence Questionnaire, developed specifically for occupational therapy students to complete in relation to their clinical placement experiences [33]. The questionnaire is a self-report measure including 41 items. Students rate their confidence from 1 (strongly disagree) to 5 (strongly agree), and scores are summed for a total score ranging from 41 to 205. The questionnaire has demonstrated high internal consistency (Chronbach’s alpha = .96), and principal component analysis, with oblique rotation, demonstrated the items formed one ‘confidence’ component scale [37]. According to the designers of the instrument, there is evidence that the tool is responsive to change in confidence levels both within a placement period (6 weeks duration tested in the study) and across placements (four placement periods).

#### Placement experience

*Student* placement experiences will be assessed using survey and focus group methods after the completion of the placement. Survey data will be collected using the 7-item SPEF-R companion instrument that asks the student to rate their experience of being oriented to the placement organisation, how welcome they felt within the workgroup, whether the clinical educator’s expectations were clear, whether they felt supported and how they felt about the evaluative experience. Responses are indicated using a 5-point Likert scale ranging from 1 = *strongly disagree* to 5 = *strongly agree*. Summed responses provide scores ranging from 7 to 35. In addition, a 14-item survey designed to assess student satisfaction with placement will be completed by students of both placement types, as the questions are generic [38]. This survey also employs a 5-point Likert scale ranging from 1 = *strongly disagree* to 5 = *strongly agree*. In addition to survey data, participating students will be invited to contribute to a focus group in which their perceptions of the meaning, value and quality of their placement experiences will be explored.

*Supervisor and educator* perceptions of SCP and TCP opportunities will be assessed using an online survey developed to mimic the placement experience questions posed to students. Responses to the questions will be indicated using a 5-point Likert scale ranging from 1 = *strongly disagree* to 5 = *strongly agree*. Educators involved in the delivery of the placement experiences will also be invited to contribute to focus groups that explore their perceptions of satisfaction and quality of the placement for early years’ occupational therapy students.

### Blinding

This is a single-blinded RCT where the assessors of the examinations are blind to the group to which students were allocated. Completed examination papers will be checked centrally to ensure no notes are included on the papers that reveal student allocation to SCP or TCP; the papers will then be distributed to markers who have no other role in the study. The examination scores will be collated and entered into the database by the project manager, who is not blinded to student group but has no interaction or history with any of the participating students. The SCP and TCP supervisors and SCP content deliverers cannot be blinded to group, nor can the student participants.

### Data monitoring and management

A data monitoring subcommittee will oversee data quality. The members are the lead chief investigator, an investigator with significant statistical expertise, an investigator representing the economic analysis team, the national project manager and the national research assistant. The project manager will undertake day-to-day management of the project. Each site will have a local research team consisting primarily of a site coordinator and research assistant. The local research teams are responsible for fidelity of delivering the intervention and collecting the data on their site. They will be provided with in-person training by the project manager at their site prior to implementing the intervention; the project manager will also maintain regular contact before, during and after the intervention to ensure the efficient collection of data according to the protocol. The local research assistants will collect and submit the data to the project manager. The national research assistant will enter the data into a project database for cleaning and analysis. Missing data will be identified centrally, and the local teams will be responsible for securing it, following ethically approved processes.

#### Statistical analyses methods

All data will be entered into a purpose-designed REDCap (https://projectredcap.org/) database, cleaned and assessed for missing data. Summary statistics will be used to describe the sample. To evaluate whether students who attend an SCP achieve non-inferior outcomes to those who attend a TCP, differences in achievement on the post-placement examination score will be evaluated using analysis of covariance (ANCOVA). A mean difference of ≥7 points on the examination score between students will be considered an educationally meaningful difference. Covariates included in the analyses will include broad placement type (vocational rehabilitation, physical rehabilitation or mental health) and any demographic variables identified as important during baseline analyses. Secondary outcomes related to whether students who attend an SCP achieve non-inferior outcomes to those who attend a TCP will be assessed using odds ratios between groups in predicting overall pass/fail grades on the SPEF-R, evaluated using binary logistic regression. Independent variables included in the analyses as possible covariates will include gender, age, broad placement type and any demographic variables identified in baseline analyses as important. Differences between SCP and TCP groups in achievement of a pass/fail grade on each of the four completed sections of the SPEF-R will be assessed using chi-square tests. Scores between SCP and TCP groups for individual SPEF-R items (scored on a 5-point ordinal scale) will be compared using a Mann-Whitney *U* test. An overall difference >1 point on the 5-point scale between groups will be considered educationally important.

To evaluate whether students and clinical educators report similar professional practice learning opportunities during SCP compared to TCP, the differences in frequencies of *not applicable* and *insufficient observation* items checked in the total SPEF-R by (1) students and (2) clinical educators will be compared using an independent samples *t* test, using 95% confidence intervals of the estimated mean difference between groups to identify differences.

To evaluate whether students who experience an SCP report non-inferior levels of confidence during placement outcomes compared to those who experience a TCP, the difference between change scores on the confidence questionnaire between SCP and TCP groups will be assessed using an independent samples *t* test. Between-group analyses of mean total score differences on this questionnaire, both pre- and post-SCP and TCP, will be determined using independent samples *t* tests. Within-group differences will be established using paired samples *t* tests.

To assess students’, educators’ and supervisors’ placement experiences, data from the SPEF-R student review of professional practice placement survey, Student Evaluation of Placement questionnaire, and Clinical Educator Evaluation of Placement questionnaire will be summarised and reported descriptively. The 5-point Likert-style item responses on each questionnaire will be reported graphically. Differences in item ratings between groups of participants will be assessed using independent samples *t* tests*.* Additional thematic analyses will be carried out on written comments made on any of the instruments in conjunction with data obtained through focus group interviews. All qualitative data will be analysed inductively to address the question of perceived placement value and quality.

#### Economic evaluation

This economic evaluation is nested within the RCT and will be closely coordinated with the purpose, data collection and statistical analysis of the RCT. The method of economic analysis will be consistent with the research question, the study perspective, the study comparators and the target population as outlined above. The choice of method for economic analysis and the time horizon will depend on the study outcomes and the decision context.

If non-inferiority of SCP compared to TCP is established in the RCT, and SCP is shown to be less expensive than TCP, then a cost-minimisation analysis will be utilised. ‘Pathway analysis’ [37] will be used to clearly specify all activities in the SCP intervention and the TCP comparator (‘who teaches what to who, when, where and how often’). If the SCP is not inferior to the TCP, but it costs more, longer term benefits will be evaluated using cost-effectiveness ratios that have pedagogical and policy meaning; for example, time to complete the occupational therapy degree and participation in the workforce. A decision threshold value will be specified in terms of what constitutes ‘value for money’ with the chosen cost-effectiveness ratios.

### Ethical considerations

To support implementation, the design of the trial ensured there was no disincentive for students to participate. All university partners have adjusted curricular requirements for the particular unit of study in which the program will be situated so that all students can be allocated to either the SCP or TCP. In addition, data collection instruments are aligned to usual educational practices and ordinary performance assessment wherever possible. These adjustments have been considered by relevant curriculum authorities in each university (for example, an Academic Program Standing Committee) to ensure pedagogical integrity. Student assignment to the placement mode will be on the basis of random allocation, regardless of their decision to take part in the study. Eligible students will be provided with the opportunity to volunteer to participate in the study; the only difference between those participating and those not will be an agreement to allow data collected as part of the placement activity to be included in the trial. Student participants will not be incentivised or compensated, and there is no advantage to participating, or disadvantage to non-participation.

Clinical supervisors, for both SCP and TCP, and the educators who help to deliver the SCP program will also be invited to participate by contributing data. The SCP supervisors and educators will be paid for their time but will not be incentivised or additionally compensated for agreeing to contribute data to the study. The TCP supervisors undertake the student supervision role within their organisations in the usual way and receive no individual payment. Some organisations charge for student placement days, and where those agreements are in place, payments to organisations will be made in the usual way and this data collected as part of the economic component of the trial.

All participants (students, clinical supervisors, simulation facilitators) can elect to withdraw from participation in the study in part or in full, but still participate in the SCP or TCP activities. All data about and from students will be labelled with the student’s institutional student number but not their name. This ensures that the various items of data, which might be submitted separately and across a span of several weeks, can be matched to particular students, but that personal details, such as the students’ names, are kept separate from the data files.

Data security during and following completion of the study will be managed in the following ways. A local research assistant based at each of the eight partner institution sites will collect the data and upload it to the overall project management team via a secure cloud storage platform. That platform is only open to academics based at Australian universities. The data will be checked and verified by a national research assistant before it is added to a central database. Each local institution will retain the original data — paper-based instruments — as per each site’s ethics requirements. As each university’s data are added to the database, those data will be deleted from the cloud server; thus the cloud server is used as a data transmission mechanism rather than as a long-term data repository.

This RCT is deemed to entail a low level of ethical risk. Students already complete professional placement activities for occupational therapy and a range of other courses, and appropriate risk management processes are already in place at each partner university. An earlier version of the SCP program has been trialled at one university with no adverse outcomes. There is no expectation that the trial will need to stop prior to conclusion.

## Discussion

The use of simulation in health-related fields like occupational therapy is not unusual, but simulation typically relates to specific activities. For example, a simulated client might be hired for students to take turns to interview, or learning activities might be held in a mock kitchen. The simulation described here is innovative in that it encapsulates a 5-day block of integrated activities, designed and delivered in a manner that aims to emulate best-practice placement experiences. This simulation is designed to replicate, and potentially replace, a short-duration traditional placement experience.

A quality traditional placement experience would comprise a range of activities for the student to participate in, roles for the student to try out and a variety of clients and professionals for the student to engage with, all in a well-supported environment [8, 9]. Despite the best of intentions, not all traditional placements will, in reality, provide this experience for students [38]. Placements might occur at a quiet time in the organisation; the supervisor may need to undertake a range of desk-top tasks in which it is difficult to engage the students; or the student’s placement experience may, for many reasons, not be the priority for the supervisor or organisation at that particular time. One important advantage of the SCP is the consistency of the content and delivery experience, and therefore the likelihood that all students will be exposed to a particular set of experiences that can lead to achieving the desired learning outcomes.

A second innovation in this study is the inclusion of the economic evaluation. The comparison of student learning and student evaluations of their experience will speak to whether the SCP is as effective as the TCP. Economic evaluation will provide valuable information about the efficiency of offering block simulation as an integral (and integrated) component of allied health courses, to partially meet the professional practice experiences required for accreditation. The economic evaluation has been carefully designed to capture all costs of providing both the SCP and the TCP. These costs are not typically transparent, and this information will be very useful to the occupational therapy profession, universities and the health and human services sector who host placements. The cost collection will also help to address the question of affordability.

Feasibility of this RCT has been enhanced through initial consultation with all universities in Australia that offer occupational therapy education [39] and in partnering with six universities in the implementation of the trial. This partnering involved collaborative agreement about what SCP to implement, how to embed the SCP across varied curricula and which measures could be included as assessment tasks within chosen units of study. This collaborative partnering will support recruitment of sufficient student participants because the only difference between participating in the trial and not participating is the question of whether required assessment data will be forwarded for the study. These factors should result in a high consent rate. There is, however, a risk related to the need to procure sufficient TCP places. Anecdotal reports from each university indicate the increasing difficulty in securing TCP places.

The current trial includes design elements that will support dissemination and knowledge transfer across occupational therapy programs in Australia. Multisite trials that aim to exert high levels of control of the trial’s implementation would typically centrally train an SCP delivery team and data collection team and dispatch them to each university to implement the SCP program in parallel with that university’s TCP program. While this might provide evidence of the effect of a highly controlled intervention, the current study is pragmatic and will provide evidence of the effectiveness of a ‘real-world’ implementation because each university is responsible for implementing the RCT, in terms of delivering the program as well as collecting the data, at their own institution. Our protocols include provision of training via a training officer, the trial manager and one of the lead investigators visiting each site to prepare the local team to deliver the SCP, administer the RCT processes and collect the data. Detailed manuals and checklists have also been prepared and ongoing support put in place to assist each site. While a pragmatic approach entails some risk to the consistency of the implementation of the RCT, these risks are offset by both the practical advantages of having local teams implement the program and the increased rapidity of knowledge translation following trial completion through ongoing use of the materials and resources developed for the project — if the hypothesis that the two placement modes are equivalent is supported by the data. Local management also reflects how a placement program would be run at a university.

### Trial status

Recruitment, and thus data collection, is ongoing at time of submission. At time of article acceptance, data collection is complete.

## Additional file

Additional file 1: SPIRIT checklist. These are our responses to the standard SPIRIT checklist. (PDF 173 kb)

## Abbreviations

SCP: Simulated clinical placement
TCP: Traditional clinical placement
